# Emerging Role of SMILE in Liver Metabolism

**DOI:** 10.3390/ijms24032907

**Published:** 2023-02-02

**Authors:** Nanthini Sadasivam, Kamalakannan Radhakrishnan, Hueng-Sik Choi, Don-Kyu Kim

**Affiliations:** 1 Department of Integrative Food, Bioscience and Biotechnology, Chonnam National University, Gwangju 61186, Republic of Korea; 2 Clinical Vaccine R&D Centre, Department of Microbiology, Combinatorial Tumour Immunotheraphy MRC, Medical School, Chonnam National University, Gwangju 58128, Republic of Korea; 3 School of Biological Sciences and Technology, Chonnam National University, Gwangju 61186, Republic of Korea

**Keywords:** SMILE, transcription factors, nuclear receptors, CREBZF, bZIP proteins, liver, metabolic pathways

## Abstract

Small heterodimer partner-interacting leucine zipper (SMILE) is a member of the CREB/ATF family of basic leucine zipper (bZIP) transcription factors. SMILE has two isoforms, a small and long isoform, resulting from alternative usage of the initiation codon. Interestingly, although SMILE can homodimerize similar to other bZIP proteins, it cannot bind to DNA. As a result, SMILE acts as a co-repressor in nuclear receptor signaling and other transcription factors through its DNA binding inhibition, coactivator competition, and direct repression, thereby regulating the expression of target genes. Therefore, the knockdown of SMILE increases the transactivation of transcription factors. Recent findings suggest that SMILE is an important regulator of metabolic signals and pathways by causing changes in glucose, lipid, and iron metabolism in the liver. The regulation of SMILE plays an important role in pathological conditions such as hepatitis, diabetes, fatty liver disease, and controlling the energy metabolism in the liver. This review focuses on the role of SMILE and its repressive actions on the transcriptional activity of nuclear receptors and bZIP transcription factors and its effects on liver metabolism. Understanding the importance of SMILE in liver metabolism and signaling pathways paves the way to utilize SMILE as a target in treating liver diseases.

## 1. Introduction

Small heterodimer partner (SHP)-interacting leucine zipper protein (SMILE), initially known as Zhangfei (ZF), is a cellular protein and a basic leucine zipper protein (bZIP) transcription factor identified as one of the coregulators involved in binding to host cell factor (HCF) required for herpes simplex virus (HSV) immediate early (IE) genes’ transactivation via virion protein VP16 [1,2]. The function of ZF was determined by the binding with HCF in a similar fashion as Luman (another cellular protein) to regulate IE genes required for the replication of HSV [3]. However, unlike other bZIP proteins, which consist of three functional regions responsible for dimerization, DNA binding, and transcriptional regulation, ZF was unable to bind to the consensus bZIP binding region and promoter regions containing the response elements for the activation of target genes [1,4]. This action was not completely understood until studies reported that bZIP proteins mediated transcription factors. Structurally, bZIP proteins consist of leucine and arginine-rich amino acid sequences responsible for binding with the major groove of DNA and facilitating the transcriptional activity [5,6,7]. A leucine in every seventh residue intervenes and forms a zipper-like structure that is important for the interaction with other bZIP proteins [8,9]. bZIP proteins are grouped into seven families of transcription factors: cAMP response element-binding protein (CREB), activating transcription factor (ATF), Jun proto-oncogene, AP-1 subunit (Jun), Fos proto-oncogene, AP-1 subunit (FOS), CCAAT/enhancer binding protein (C/EBP), Avian musculoaponeurotic fibrosarcoma (Maf), and the PAR subfamily [9]. These families of bZIP transcription factors are classified based on the protein homology and DNA binding specificity [8]. bZIP proteins consist of a consensus quintet sequence, which is important for DNA binding NXXAAXX (C/D) R, where X represents the amino acid sequence. The N residue in the bZIP region bends the protein to the major groove of DNA [9]. Among the bZIP amino acid residues, the asparagine (Asn), arginine (Arg), leucine (Leu), and isoleucine (Ile) residues are considered important in DNA binding [8]. Likewise, the N residue is considered important in DNA binding [1]. Data from the SWISSPROT database were used to understand the role of the N- residue along with the bZIP protein CHOP, encoded by GADD153, which lacks the N- terminus and does not bind to DNA; however, it does bind to DNA as a homodimer [10]. Studies have shown that ZF also lacks the N residue, and conformational changes caused by a lack of an Asn residue suggest that ZF cannot bind to DNA [3].

Later, ZF was identified as the orphan nuclear receptor SMILE, which acts as a coregulator for various nuclear receptors (NRs) and other transcription factors [11]. NRs are a superfamily of receptors that includes orphan nuclear receptors, which lack known endogenous ligands [12]. The action of NRs initiates upon the binding of a ligand to the ligand-binding (LBD) domain, and the activity involves the five functional domains of the NR. Each domain exhibits different functions in various cellular activities [13]. Since NRs act as transcription factors, they are responsible for regulating the expression of target genes via the action of coactivators and corepressors. Coactivators and corepressors binding to the NR and bZIP transcription factors are considered significant in regulating gene expression [14]. When SMILE binds to specific nuclear receptors, in the absence of ligands, it represses the activity of the transcription factors, including constitutive androstane receptors (CAR), retinoid X receptors (RXR), and estrogen-related receptor gamma (ERR-γ) [11,15]. Understanding the effect of SMILE on NRs, bZIP proteins, and other transcription factors involved in metabolism and signaling processes in the liver and other organs and tissues by regulating the expression of specific target genes is necessary.

Interestingly, SMILE and its isoform CREBZF have been shown to be an inducible transcriptional corepressor and a downstream mediator of endocrine and metabolic signals, such as insulin, drugs like metformin, and bile acids, in the liver [16,17,18]. Thus, SMILE is considered a critical corepressor, contributing to the pathogenesis of various liver diseases, including diabetes, non-alcoholic fatty liver disease (NAFLD), hyperglycemia, and cystic fibrosis [17,19]. SMILE has also been shown to play a role in reproduction and cancer [20,21,22,23]. In recent times, SMILE was shown to suppress the transcriptional activity of many nuclear receptors such as liver X receptor (LXR), pregnane X receptors (PXRs), farnesoid X receptor (FXR), CAR, hepatocyte nuclear factor 4 (HNF4), and estrogen-related receptors (ERRs) and other transcription factors such as CREB, cyclic AMP-responsive element-binding protein H (CREBH), steroid regulatory element binding protein-1c (SREBP-1c), forkhead box protein O1 (FoxO1), and signal transducer and activator of transcription 3 (STAT3), which are implicated in hepatic glucose, lipid, and bile acid metabolism and bacterial infections [11,24,25,26]. Therefore, SMILE is a crucial inducible corepressor and can be considered an important target in treating metabolic diseases.

## 2. SMILE Background

**Figure 1:**
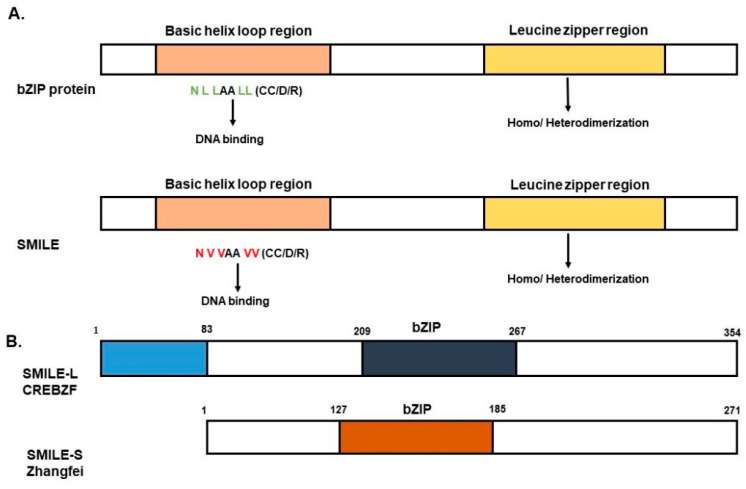
SMILE structure. (**A**). Small heterodimer partner-interacting leucine zipper (SMILE) is a bZIP transcription factor. The sequence of NLLAALL, where L represents leucine, is important in DNA binding. The lack of the N residue and the change of leucine to valine results in a conformational change that is the difference between other bZIP proteins and SMILE, where SMILE lacks the ability to bind to the DNA as a homodimer. (**B**). SMILE has two isoforms, a long form known as SMILE-L/CREBZF and a short isoform known as SMILE-S/Zhangfei (ZF), which are formed by the alternative initiation codon. SMILE-L/CREBZF has the N-terminus region that spans around 83 amino acids, similar to the bZIP CREBH family. The numbers indicate the amino acid number.

SMILE is a member of the CREB/ATF family of bZIP proteins [27]. The function of SMILE was determined when SMILE counteracted the repressive action of SHP in estrogen receptor (ER)-mediated transcription. This report showed that SMILE could be a potential corepressor that acted as a repressive protein for NR-mediated transactivation. The interaction between SHP and SMILE was determined using a yeast hybrid system [28]. Structurally, SMILE is a 354-amino-acid-long protein in which a region from amino acids 113 to 202 is responsible for the corepressor activity by interacting with the LBD regions of the NRs. SMILE competes with coactivators in the binding of NRs. Structurally, SMILE has two isoforms as a result of alternative splicing. The long isoform (SMILE-L/CREBZF) has an extra 83-amino-acid-residue spanning region compared with the short isoform (SMILE-S/Zhangfei) and has a similar structure to the bZIP protein CREB [11]. Although SMILE has the ability to homodimerize similar to bZIP proteins, it cannot bind to DNA as a homodimer and differs from other bZIP transcription factors by specific structural conformation changes such as lacking a N-terminus and the change of leucine residue repeats [2]. The change of leucine to valine amino acid residue and the lack of the N-terminus are responsible for identifying SMILE as a corepressor (Figure 1) [11]. SMILE acts as a coactivator or corepressor depending on its interaction with specific transcription factors. For instance, by forming a heterodimer complex with activator transcription factor 4 (ATF4), it augments the activation of the CRE reporter, whereas by forming a complex with VP16 and CREB3, it functions as a corepressor in HCF activation [29]. Interestingly, hepatic SMILE is an inducible corepressor and is regulated via the signaling pathways mediated by several factors such as curcumin, ursodeoxycholic acid (UDCA), epigallocatechin gallate-1 (EGCG), anti-diabetic drugs like metformin, and insulin [25,30,31]. The mediated activation of these factors by SMILE regulates cellular processes such as metabolism, development, and homeostasis by suppressing the transactivation of NRs and other transcription factors. The action of SMILE in the mentioned cellular process involves specific modes of action.

## 3. Mode of Action of SMILE in Target Gene Regulation

**Figure 2:**
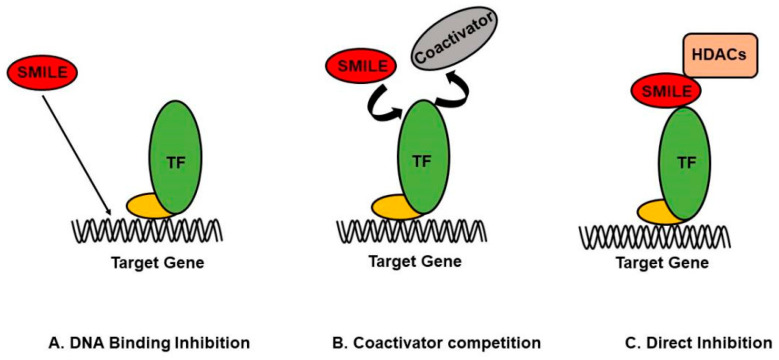
Modes of action of SMILE (**A**). SMILE, as a transcriptional corepressor, binds directly to DNA and inhibits target gene transcription. (**B**). Target gene expression is regulated by the corepressor SMILE and several coactivators competing to bind to the transcription factors. (**C**). SMILE recruits enzymes such as histone deacetylases (HDACs) to inhibit the transcription of target genes upon binding to the transcription factor as a complex.

SMILE plays a major role in regulating the activity of NRs and bZIP transcription factors by various modes of action (Figure 2). Firstly, SMILE can direct DNA binding and thereby inhibit the transactivation of the transcription factors such as HNF4, LXRs, CAR, ERR-γ, and runt-related transcription factor 2 (RUNX2). It is necessary to understand the role of SMILE in various transcription factors by direct DNA binding inhibition. The action of SMILE on peroxisome proliferator-activated receptors (PPARs), ligand-dependent transcription factors with three subtypes, i.e., PPAR-α, γ, β/δ, exhibits different functions [32,33]. PPARs are similar to steroid and thyroid hormone receptors and form a heterodimer with Retinoid X receptor (RXR). PPARs are stimulated in the presence of lipophilic ligands by forming a complex with coactivators and regulate gene expression. However, in the absence of ligands, a corepressor binds to the PPARs and RXR complex and blocks gene expression by direct DNA inhibition [34]. PPAR-α is responsible for reducing triglyceride levels and is involved in regulating energy homeostasis. Likewise, PPAR-γ regulates glucose metabolism and adipocyte differentiation [35]. PPAR-γ binding to the adiponectin (Adipoq) promoter regulates adipocyte differentiation and leads to fat accumulation. SMILE induced by tunicamycin, a key ER-stress inducer, directly interacts with PPAR-γ and inhibits binding to the Adipoq promoter, thus reducing the expression of Adipoq [36]. PPAR-β/δ is involved in fatty acid metabolism [37]. Another example of the DNA binding inhibition of SMILE is the interaction with STAT-3 via IL-6-mediated DNA binding inhibition, which results in inhibiting the expression of target genes responsible for hepcidin production in iron metabolism [38]. Likewise, SMILE inhibits the DNA binding ability of suppressor of mothers against decapentaplegic (SMAD) via the BMP-6 pathway by directly binding to the promoter and attenuating the expression of hepcidin [39]. DNA-binding inhibition of BMP-2 is an important regulator of osteoblast regulation, bone repair, and bone development induced via ER stress. SMILE regulates osteoblast differentiation by directly inhibiting RUNX2, thereby inhibiting the ability of RUNX2 to bind to osteocalcin (OC) promoters [40]. Secondly, through the coactivator competition mode of action, SMILE is reported to inhibit the transactivation of many transcription factors, including HNF-4α, CREB, and FoxO1. HNF-4α is mainly expressed in the liver and gastrointestinal tract, is a strong transcription factor belonging to the steroid hormone nuclear receptor superfamily, and binds to the DR-1 element of target gene promoters as a homodimer [41]. In glucose metabolism, inhibition is mediated by SMILE competing with the coactivator peroxisome proliferator-activated receptor γ coactivator-1α (PGC-1α), as HNF-4α transcriptional activity is regulated by the coactivator PGC-1α, which downregulates gluconeogenic gene expressions [11,42].

Many transcription factors regulate gene expression by binding with coactivators, and SMILE possibly inhibits the transactivation of these transcription factors by competing with specific coactivators. Another mode of action of SMILE is direct repression by recruiting histone deacetylases (HDACs) to suppress the target gene activation mediated via transcription factors [43]. The role of HDACs in SMILE-mediated gene repression was reported when the HDAC-specific inhibitor trichostatin-A inhibited the corepressor action of SMILE on NRs and implied that HDAC recruitment is important for SMILE activity. Moreover, the repression activity of SMILE mediated by HDACs is very specific to transcription factors [11]. Many HDACs can interact with SMILE and inhibit the transcriptional activity of SMILE; however, only a limited number of studies have revealed this mode of action. One such example is the action of SMILE on ERR-γ by recruiting sirtuin type 1 (SIRT-1), a group of histone protein deacetylases that negatively regulate transcription by direct interactions with the target genes [15]. The SMILE-mediated direct inhibition of various transcription factors by recruiting HDACs needs more analyses and studies to understand this specific mode of action.

## 4. The Role of SMILE in Regulating Liver Metabolism via Various Signaling Pathways and Transcription Factors

**Figure 3:**
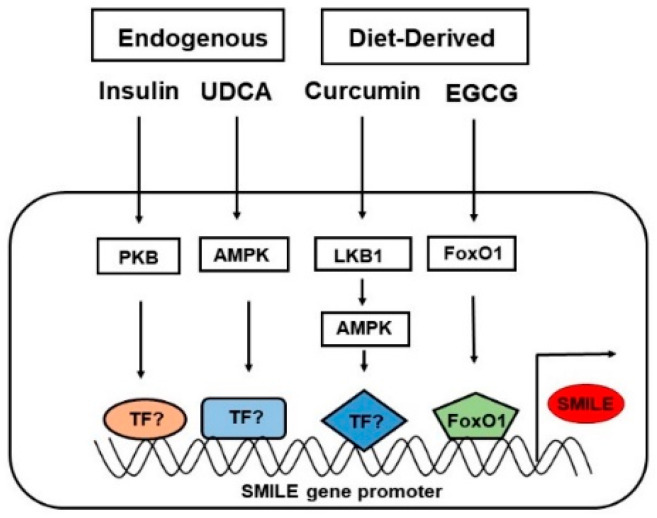
SMILE, an inducible transcriptional corepressor. Many inducible stimuli such as insulin, curcumin, UDCA, and EGCG are known inducible factors that activate SMILE expression via the respective pathways. The endogenous inducers insulin and UDCA mediate SMILE expression via the PKB and AMPK pathways, respectively. Diet-derived curcumin induces SMILE expression via LKB1, which in turn activates AMPK signaling. EGCG, also as a diet-derived stimulus, mediates expression via FoxO1, which acts as a known transcription factor for SMILE expression. SMILE binds to the transcription factors and inhibits the target gene expression, acting as an inducible transcription factor.

**Figure 4:**
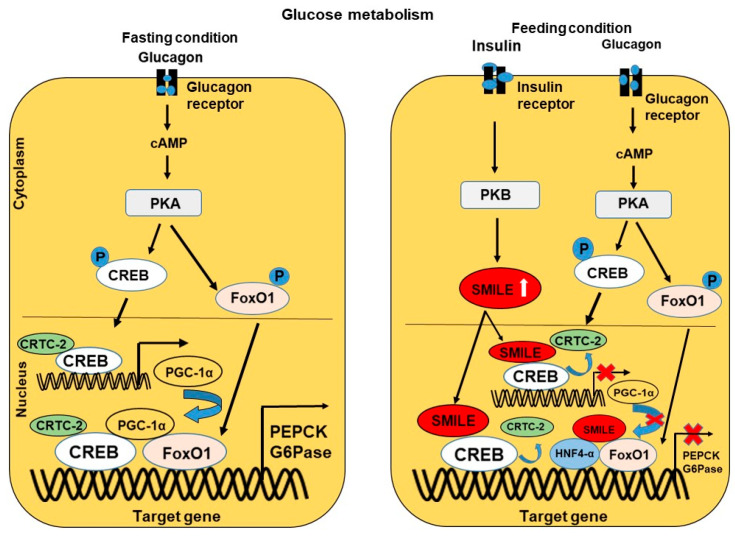
The role of SMILE in regulating glucose metabolism. SMILE is an inducible transcription factor that binds to various transcription factors, such as FoxO1, CREBH, STAT-3, nuclear receptors, SREBP-1c, CREB, and HNF4α, which are activated by the signals of various liver metabolic pathways, including glucose, lipid, and iron. Glucose metabolism involves fasting and feeding conditions and involves the action of the hormones insulin and glucagon. These hormones regulate the metabolic signaling pathways via PkB/Akt and activate the transcription factors through phosphorylation, resulting in the regulation of gluconeogenic genes. During fasting, glucagon activates cAMP, which in turn activates the transcription factors CREB and FoxO1 via the PKA pathway. CREB, along with the binding of the coactivator CRTC-2, activates the transcription of PGC-1α, which acts as a coactivator for other transcription factors. In contrast, during feeding conditions, insulin induces SMILE via the PKB pathway and inhibits the action of CREB by competing with CRTC-2, inhibiting the action of PGC1α on other transcription factors and inhibits the transcription of gluconeogenic genes involved in glucose metabolism.

**Figure 5:**
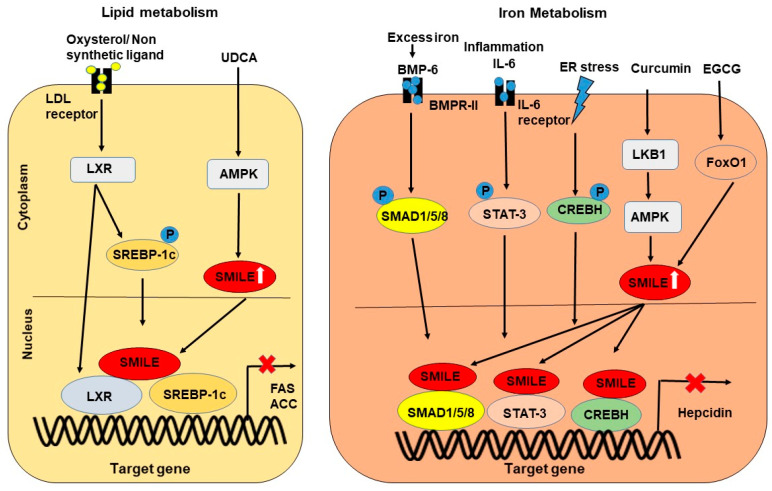
The role of SMILE in regulating lipid and iron metabolism- During lipid metabolism, oxysterols binding activates the transcription factor SREBP-1C via LXR activation, and the action of UDCA induces SMILE via the AMPK pathway, which inhibits the transcription of ACC and FAS (left panel). Curcumin and EGCG induce the expression of SMILE. SMILE inhibits the action of SMAD1/5/8, which are activated due to excess iron levels via BMP-6 and STAT-3 (right panel). BMP-6 and STAT-3 are activated due to inflammation and ER stress induced by CREBH. Inhibition of the transcription factors by SMILE inhibits genes responsible for hepcidin production.

SMILE has a significant role in regulating genes involved in the metabolism of glucose, lipid, iron, and bile acids in the liver [11,28]. Since SMILE is believed to bind to specific transcription factors such as NRs and bZIP proteins, which are responsible for the transcriptional activity of metabolic genes, SMILE might be involved in the repression of these genes in liver-mediated diseases. Inducible factors, including insulin, EGCG, curcumin, and UDCA, activate the expression of SMILE, and thus SMILE is an inducible transcriptional corepressor (Figure 3). Upon the stimulation of SMILE, SMILE inhibits target gene expression by various modes of action, as mentioned above, in response to metabolic signaling pathways and transcription factors (Figure 4 and Figure 5). The action of SMILE in liver metabolism and related transcription factors is as follows.

### 4.1. Glucose Metabolism and Insulin Resistance

Many NRs and bZIP transcription factors are involved in regulating liver-mediated metabolic signals. Glucose metabolism is a common metabolic pathway in the liver [44]. Blood glucose homeostasis and the underlying metabolic signaling pathways are very important in controlling specific liver diseases related to glucose levels [45,46]. The major coactivators involved in glucose metabolism are PGC-1α and CREB-regulated transcription coactivator-2 (CRTC-2) (Figure 4, left panel). The action of these coactivators is controlled by insulin and glucagon during feeding and fasting conditions, respectively [47,48,49]. During a fasting condition, glucagon binds to the glucagon receptor and activates the protein kinase A (PKA) signaling pathway, which is involved in regulating the expression of gluconeogenic genes by the transcription factor CREB [50]. The interaction of CREB with the coactivator CRTC-2 induces the expression of PGC-1α and ERR-γ [47,51,52]. PGC-1α, a coactivator protein, is responsible for acting as a coactivator for the transcription factors HNF-4α and FoxO1 and the transcription of gluconeogenic genes [53,54].

In contrast, during a feeding condition, studies have reported that insulin represses the activity of the transcription factors, regulated via the glucagon/PKA axis, which induces the coactivators [55,56,57]. Later it was discovered that insulin suppression was related to the SMILE corepressor activity via a mouse model (Figure 4, right panel). This study revealed that SMILE is an insulin-inducible corepressor that competes with CRTC-2 in the binding of CREB and inhibits the expression of PGC-1α, ultimately inhibiting PGC-1α binding to other transcription factors and thereby preventing the expression of up-regulated gluconeogenic genes such as glucose-6-phosphatase (G6Pase) and phosphoenolpyruvate carboxykinase (PEPCK) [58]. Transcription factors, such as HNF4α and ERR-γ, also show inhibitory effects related to glucose signaling by interacting with SMILE [11]. Additional experimental models and evidence will provide future insight into considering SMILE a novel target for treating glucose metabolism-mediated disease, including diabetes, hyperglycemia, and hypoglycemia.

### 4.2. Lipid Metabolism

The major transcriptional regulators involved in lipid metabolism are LXR, SREBP-1c, PPAR-γ, and ERR-γ, and they are regulated via the lipogenic pathways [59,60,61,62]. LXR and PPAR-γ are activated in response to cholesterol metabolites such as oxysterols and synthetic non-steroids. ERR-γ is an NR that is reported to be the regulator of LIPIN1. LIPIN1 is a gene that encodes the lipogenic protein phosphatidic acid phosphatase Lipin-1. Studies on lipid metabolism related to ERR-γ and the activation of Lipin-1 report that ERR-γ induces the expression of Lipin-1 by interacting with the coactivator PGC-1α. SHP inhibits the expression of LIPIN1 by competing with the coactivator for binding to ERR-γ [63]. Since SHP is related to SMILE, SMILE might also be involved in Lipin-1-related lipid synthesis via ERR-γ transcriptional activity.

However, there is no direct evidence of SMILE activity and ERR-γ mediated lipid synthesis. Similar to ERR-γ, another transcription factor, SREBPC-1c, a major lipid synthesis regulator, is activated via LXR and the cholesterol byproduct oxysterols [64,65]. Evidence of SMILE interaction with the transcriptional activity of SREBPC-1c related to lipid metabolism is reported to be caused by the action of antagonists of LXR such as ursolic acid (UA). UA is a plant compound that reduces the expression of LXR and the promoter activity of SREBP-1c and decreases the hepatic lipid content. Thus, SMILE is reported to be a possible corepressor that inhibits the transcriptional activity of target genes such as fatty acid synthase (FASN) and acetyl-CoA carboxylase (ACC) responsible for lipid metabolism mediated via repression of the LXR and SREBP-1c transcription factors (Figure 5, left panel). These results also indicate that UA may act as a potential factor that induces the inhibition of the transcriptional activity of SREBPC-1c and LXR via SMILE [66]. UA is an antagonist of LXR and increases SMILE activity via AMP-activated protein kinase (AMPK) phosphorylation in hepatic cells. However, UA decreases the activity of SMILE and reverses cholesterol transport in intestine cells [66]. SMILE-L/CREBZF regulates insulin-mediated lipogenesis via the insulin/AKT signaling pathway. Insulin-induced gene-2 (insig-2) downregulation induces SREBP-1c to mediate lipogenesis. This mechanism is unclear, with the suppression of insig-2 occurring even during refeeding conditions. Later it was reported that insulin-mediated inhibition of insig-2 via SMILE-L/CREBZF. SMILE-L/CREBZF directly binds to insig-2 by cooperating with activating transcription factors (ATFs) [67]. To confirm the SMILE-L/CREBZF-mediated repression of insig-2, liver-specific knockout of CREBZF induced insig activation and suppressed lipogenic processes. Moreover, the knockdown of CREBZF resulted in hepatic steatosis. Additionally, CREBZF expression was increased in genetically modified obese mice. In contradiction to the previous reported studies, CREBZF is reported to be involved in mediating hepatocyte injury and affects the liver of a diet-induced mouse model of NASH. Downregulation of CREBZF results in liver fibrosis, inflammation, and liver injury in the diet-induced mice model. The mechanism of this condition in the mice is the inhibition of miR-6964-3p by CREBZF, which results in the secretion of osteopontin, a regulator of fibrosis. This study indicates that CREBZF can also act as a negative mediator in fatty liver disease [68]. These observations indicate the importance of SMILE-L/CREBZF in lipogenesis mediated via insulin and provide new insight into the insulin-mediated lipogenesis mechanisms [67].

To further analyze the activity of SMILE in hepatic lipid metabolism, bile acid was focused on due to its major role in triglyceride homeostasis and metabolism. One such bile acid derivative is UDCA, which has beneficial effects on liver-related diseases through its essential role in the phosphoinositide 3-kinase (PI3K)/AKT/nuclear factor erythroid-2-related factor 2 (Nrf2) pathway. However, UDCA treatments in animal models have been shown to repress the action of lipogenic genes through interactions with SMILE. Experimental evidence suggests that SMILE inhibits the lipid metabolism-related receptor LXR directly and with external compound interactions such as UDCA treatments via the inhibition of the transcriptional activity of LXR [25]. In addition, other NRs such as PPARs are known to regulate lipid metabolism in the liver, but the action of SMILE as a corepressor of PPAR activity is unknown. These consistent results indicate that the action of SMILE via external ligands is liver-specific through the interactions with NRs, and this interaction can be a potent therapeutic target for treating various lipid metabolic diseases such as hepatic steatosis, NAFLD, and hyperlipidemia.

### 4.3. Iron Metabolism and the Inflammatory Response

SMILE has also been reported to be involved in regulating iron metabolism. Studies have shown that SMILE acts as a corepressor for NR- and bZIP-related transcriptional activity in glucose and lipid metabolism by binding to endogenous ligands, either external or internal, but the complete activity of NRs or other transcription factors combined with the action of corepressors in regulating iron metabolism is not well understood. Recently, the regulation of iron metabolism via inflammatory response signals, excess iron levels, ER stress, IL-6 activated transcription factors, and SMILE-mediated transcriptional activity was shown to act by inhibiting hepcidin production [38,69]. Transcription factors such as STAT-3, SMADs, and bZIP transcription factors are also reportedly involved in hepcidin production [70,71]. These transcription factors regulate the hepatic hormone hepcidin, which is responsible for regulating iron homeostasis (Figure 5, right panel). Previous studies have reported that SHP, a corepressor of NRs, repressed the activity of hepcidin via the bone morphogenetic protein 6 (BMP-6)/SMAD signaling pathway [72]. The interaction of SMILE-L/CREBZF with SMAD1, SMAD5, and SMAD8, which are mediated by BMP-6, was identified by a yeast two-hybrid system using the Mad homology-2 domain of SMAD2 as bait. The interaction was further confirmed by immunoprecipitation in a human prostate cancer cell line revealing that SMILE-L/CREBZF is a key regulator in SMAD/1/5/8-mediated transactivation [73].

Likewise, hepcidin regulation via STAT-3 has been shown to be repressed by SMILE through direct interactions resulting in an abnormal iron deficiency in the blood called hypoferremia. SMILE is induced by various naturally occurring compounds, such as curcumin and EGCG, which stimulate the expression of SMILE in various cells, including hepatocytes, endothelial cells, and cancer cells [74]. Studies on EGCG have shown that EGCG induces the activity of SMILE by activating the AMPK pathway through liver kinase B1 or reactive oxygen species (ROS)-dependent calmodulin-dependent protein kinase kinase-β (CAMKKβ). EGCG has been shown to increase the expression of FoxO1 and SMILE in hepatic cells [38]. Through the activation of FoxO1 by EGCG, SMILE inhibits STAT-3 and suppresses the secretion of hepcidin, which is important for maintaining iron levels in the liver [38]. Another transcription factor involved in iron metabolism is the bZIP protein CREBH, which is highly expressed in the liver and intestine and is involved in regulating the expression of hepcidin [75]. CREBH is an ER-stress-mediated transcription factor, and upon external stimuli, ER stress activates CREBH, and it binds and transactivates the hepcidin promoter [69]. The expression of hepcidin is mediated by CREBH and is inhibited by curcumin, which induces SMILE [76]. SMILE binds with CREBH and represses hepcidin. SMILE acts as a negative regulator for hepcidin overproduction and affects iron homeostasis in the liver by causing anemia of inflammation, and hepcidin deficiency leads to hemochromatosis [76]. Targeting the ER response pathway with SMILE as a corepressor would give insight into controlling hepcidin production through CREBH. Evidence of the involvement of SMILE in iron metabolism is limited; however, targeting SMILE in iron metabolism will provide insight into understanding the mechanisms of SMILE in the context of iron homeostasis, which will be useful in overcoming iron metabolism related diseases, such as anemia and hypoferremia.

## 5. Non-Hepatic Functions of SMILE

Additionally, SMILE/CREBZF/Zhangfei is reported to have significant roles in non-hepatic cellular functions. Although the role of SMILE is not fully understood, studies related to it are not limited to the liver. The other isoform of SMILE, CREBZF/Zhangfei, is known to be involved in reproduction, cancer, apoptosis, and other cellular functions [77,78,79]. A few studies have reported on the expression of CREBZF in reproduction biology. Among the two CREBZF isoforms, SMILE and Zhangfei, SMILE is predominantly reported to be expressed in the uterus and is localized to luminal and glandular epithelial cells [20]. The expression of SMILE is estrus cycle-dependent and is elevated during ovulation. Females experience an increase in SMILE during embryo development, which indicates that SMILE may influence early embryo production [20,77]. SMILE has been reported to upregulate estrogen; however, the complete role of SMILE/CREBZF in female reproduction is not well understood and needs more evidence and studies. CREBZF/SMILE also plays an important role in male testosterone production. Since SMILE is dominantly expressed in reproductive tissues, the relationship between the reproductive hormones human chorionic gonadotropin (hGC)/luteinizing hormone (LH) and CREBZF/SMILE was studied. This study reported that CREBZF/SMILE promoted hGC-induced testosterone production in mouse Leydig cells by acting on the orphan nuclear receptors Nr4a1 and Nr5a1 and thereby increasing the expression of steroidogenic genes [80]. The action of SMILE on the androgen receptor (AR), a key receptor in prostate cancer regulation, is important in the transactivation of androgenic genes [31]. The proliferation of prostate cancer is inhibited by metformin, an antidiabetic drug. Studies have reported that metformin induces SMILE, which acts as a corepressor of AR and inhibits the proliferation of prostate cancer cells via androgen-dependent growth. The role of CREBZF/SMILE in reproduction is not well understood since the signaling pathways and the complete mechanism are not well studied.

CREBZF/SMILE is also reported to play a role in cancer cells and apoptosis. CREBZF/SMILE is involved in apoptotic functions, and reports have suggested that CREBZF/SMILE may be involved in pro-apoptotic activities during cell apoptosis via the extracellular signal-regulated protein kinase (ERK1/2) and mammalian target of rapamycin (mTOR) pathways [23]. The relationship between apoptosis and CREBZF/SMILE was studied, and it was reported that in regulating apoptosis, CREBZF increases the expression of BCL2-associated X protein (BAX) and cleavage of caspase-3 and downregulates the expression of B-cell lymphoma 2 (BCL-2). CREBZF/SMILE is also reported to be involved in cancer cell metabolism since CREBZF/SMILE is considered to be a positive regulator of the tumor suppressor p53. CREBZF/SMILE interacts with HES-related family BHLH transcription factor with YRPW motif 1 (HEY1), a nuclear protein belonging to the HESR family of basic helix loop type transcriptional repressors, which were reported to be involved in indirectly activating p53. Furthermore, the depletion of CREBZF/SMILE reduced the activity of p53 and inhibited the HEY1-mediated transactivation of p53. These results suggest that CREBZF/SMILE is a positive regulator of tumorigenesis [21]. SMILE also plays a key regulator role in skin cancer. The expression levels of SMILE were reported to be downregulated in human melanoma cells according to biopsy studies. The action of SMILE in melanogenesis is related to the microphthalmia-associated transcription factors (MITF) that are regulated by the α-melanocyte-stimulating hormone responsible for melanogenesis. In B16F10 mouse melanoma cells during melanogenesis, SMILE was regulated via the α-MSH/protein kinase A/cAMP pathway and suppressed the MITF-mediated transcriptional activity of melanogenic genes. SMILE acts as a corepressor for the transcriptional activity of cAMP response-binding proteins, thereby inhibiting melanin production in melanocytes. Therefore, this study reports that SMILE affects the cAMP signaling pathway and regulates the melanogenic transcriptional activity [81].

BMP-2, induced by mild ER stress and responsible for osteoblast differentiation, mediates osteocalcin gene expression via RUNX2, ATF-6, ALP, and Dlx-5, which are regulated by curcumin treatment. Curcumin enhances osteoblast differentiation. Interestingly, the repressive action of SMILE/CREBZF in curcumin-treated osteoblast differentiation was downregulated [40,82]. Previously, tunicamycin, an ER-stress inducer, was reported to upregulate the expression of SMILE/CREBZF and prevent BMP-2-mediated osteoblast differentiation. This result shows that the regulatory action of SMILE/CREBZF differs in response to various factors [40,83]. SMILE/CREBZF is also involved in bone and fat formation, which is regulated via neurological signals. PGE-2/EP-4 skeleton interoception regulates bone homeostasis, and as the signals increase, hypothalamic neuropeptide Y (NPY) induces the lipolysis of adipose tissue for osteoblast bone formation, which in turn disrupts bone homeostasis. Interestingly, SMILE induced in the hypothalamus binds with pCREB and to the promoter of NPY, which inhibits the expression of NPY. The downregulation of the expression of NPY through the decrease in regulation by neuroendocrine signals results in fatty acid lipolysis for bone formation [84]. All of these results show that the non-hepatic functions of CREBZF/SMILE need more in-depth analyses.

## 6. Conclusions

Recent findings on the transcriptional corepressor SMILE suggest that it can regulate liver metabolic signals and pathways involved in glucose, iron, and lipid metabolism. SMILE is also involved in multiple key biological functions, such as reproduction biology, apoptosis, and cancer. SMILE acts by binding to the NRs and bZIP transcription factors to regulate target genes by the action of DNA binding, target genes, and direct inhibition. Considering the importance of SMILE as a corepressor in the liver and other tissues and organs, targeting SMILE will provide a novel therapeutic approach for various liver-mediated metabolic diseases and other pathological conditions involving the transcriptional regulation of vital genes. Thus, the regulation of SMILE in the glucose, lipid, and iron metabolic pathways has a major beneficial role in maintaining blood glucose levels, lipid synthesis, and iron homeostasis, which control metabolic diseases such diabetes, hyperglycemia, hypoglycemia, hepatic steatosis, NAFLD, hyperlipidemia, anemia, and hypoferremia. However, the number of studies related to the interaction of SMILE in recruiting other repressive enzymes, coactivator binding, and direct DNA interactions is limited. SMILE is an inducible transcriptional corepressor; however, the complete mechanism and the regulatory pathway that induce the expression of SMILE are not completely understood. SMILE has a significant role in regulating metabolic signaling and hepatic disease-related genes. SMILE also plays considerable roles in non-hepatic functions such as reproduction, apoptosis, and cancer. SMILE may represent an attractive therapeutic target for various metabolic diseases and warrants more in-depth studies.

## Data Availability

Not applicable.
